# The influence of cognitive schemas on the mixed anxiety-depressive symptoms of breast cancer patients

**DOI:** 10.1186/s12905-020-00898-7

**Published:** 2020-02-24

**Authors:** Ana Cristina Bredicean, Zorin Crăiniceanu, Cristina Oprean, Ioana Alexandra Riviș, Ion Papavă, Ica Secoșan, Mirela Frandeș, Cătălina Giurgi-Oncu, Daciana Grujic

**Affiliations:** 1 Department of Neuroscience, “Victor Babeș” University of Medicine and Pharmacy, Timișoara, Romania; 2 Department of Plastic and Reconstructive Surgery, “Victor Babeș” University of Medicine and Pharmacy Timișoara, Timișoara, Romania; 3 “Pius Brânzeu” County Emergency Hospital, Timișoara, Romania; 4 “Victor Babeș” University of Medicine and Pharmacy, Timișoara, Romania; 5 Oncohelp Centre, Timișoara, Romania; 6 “Carol Davila” University of Medicine and Pharmacy, Bucharest, Romania; 7 Department of Functional Sciences, “Victor Babeș” University of Medicine and Pharmacy, Timișoara, Romania

**Keywords:** Breast cancer, Cognitive schemas, Anxiety, Depression, Mastectomy, Breast reconstruction

## Abstract

**Background:**

The surgical treatment of breast cancer involves various psychological consequences, which differ according to individual characteristics. Our study aimed to identify the role that cognitive schemas had in triggering anxiety and depressive symptoms in patients diagnosed with breast cancer that underwent oncological and plastic surgery treatment.

**Methods:**

64 female patients, diagnosed with breast cancer from an Oncology and Plastic Surgery Hospital, were selected to participate in this study between March–June 2018. They were divided into two groups: I. 28 patients who underwent mastectomy surgery; II. 36 patients, who required mastectomy and, subsequently, also chose to undergo breast reconstruction surgery. For the purposes of evaluating a possible change in mental health status, we employed two assessment scales: the Young Cognitive Schema Questionnaire - Short Form 3 (YSQ-S3) and the Romanian version of the Depression Anxiety Stress Scale – 21 (DASS-21R).

**Results:**

Participants who underwent mastectomy and subsequent breast reconstruction surgery employed cognitive schemas that did not generate symptoms of depression or anxiety. In contrast, the cognitive schemas found in women who refused reconstructive breast surgery were significantly correlated with the presence of anxiety-depressive symptoms. The cognitive schema domain of ‘disconnection and rejection’ correlated uncertainly with the presence of anxiety-depressive symptoms for the group with breast reconstruction (Spearman’s *ρ* = 0.091, *p* = 0.644), while for the other group the correlation was moderate-strong (Spearman’s *ρ* = 0.647, *p* <  0.01). Negative emotional schemas were significantly correlated with the presence of anxiety-depressive symptoms (Spearman’s *ρ* = 0.598, *p* <  0.01) in the group of participants without reconstructive surgery.

**Conclusion:**

A correct identification of dysfunctional cognitive schemas and coping mechanisms at the commencement of the combined treatment in breast cancer patients could serve as an indicator for the evolution of their mental health, therefore assisting professionals in establishing the most suitable psychological, psychotherapeutic and psychiatric intervention plan.

## Introduction

Breast cancer appears to be the leading cause of death in women, as it represents the most common type of cancer diagnosed in this demographic category [1, 2]. It causes a multitude of difficulties within the patients` families, their social system, as well as for the wider public health system. The treatment is complex and involves three main types of interventions: surgery, chemotherapy, and radiotherapy [3]. Long-term hospitalization, elaborate medical interventions, and side effects of oncological therapy represent a vulnerable combination for the unfolding of psychiatric symptoms in the area of anxiety and depression [4]. In Romania, more than 6000 new cases of breast cancer are reported each year, while the percentage of depressive spectrum disorders varies between 10 and 25% [5]. Depressive symptoms in women with breast cancer are reported extremely often, because of a multitude of factors, such as: the manifestation of the disease itself, pain and fatigue, the change in self-image, the impact on their sexual intimacy, the lack of expected support, the fear and the need to adjust to the new situation. The patient’s life changes dramatically, immediately following diagnosis, as they need to reconsider their priorities, engage in a new set of behaviors, accept limitations, and restructure their core beliefs [6].

Depression is described as a feeling of marked sadness, accompanied by feelings of futility and guilt. Furthermore, the person will display social withdrawal or isolation, hopelessness, lack of joy, as well as significant changes in appetite and sleep [7, 8]. Most oncology patients tend to experience these negative feelings at some point in the course of a malignant pathology. The link between depression and cancer is bidirectional, since it is possible for depression to be triggered by receiving the diagnosis of cancer, but also to occur as a consequence of its recommended treatment [9, 10]. Furthermore, people who experienced depression appear to be at a higher risk of developing a malignancy [11].

Alongside depression, anxiety may also occur, with the two disorders representing the main psychiatric symptoms experienced by breast cancer patients [12–14]. Anxiety is usually expressed through uncontrollable worry, intense fear that takes the form of panic attacks, upsetting dreams, or flashbacks. Patients describe an uncontrollable fear, restlessness, hyper-vigilance, insomnia, dyspnea, tachycardia, numbness, fatigue, or muscle tension. Anxiety may ensue after receiving the diagnosis of breast cancer, during the treatment phase, or later, in the disease-monitoring stage [15].

Anxiety, when associated with a neoplasm diagnosis, can decrease the pain threshold, interfere with the individual’s sleeping pattern, can trigger digestive symptoms, thus interfering significantly with the person’s quality of life. However, the theory that suggests people who have found a purpose in life tend to express a lower subjective sense of anxiety and fear of death was advanced by certain authors [16].

Many clinical trials have emphasized a correlation between breast cancer and mixed anxious-depressive symptomatology [17]. Based on the type of medical intervention employed, these symptoms may be amplified. Research shows that, in the first year after an early-phase breast cancer diagnosis, up to 50% of women may experience anxiety and/or depression. This percentage tends to decrease during the following years; however, up to 15% of women may still experience these symptoms in the fifth year after diagnosis [18]. Symptoms that occur in relation with the oncological treatment, such as fatigue and pain, increase the risk of developing anxiety and depression. In addition, treatments, such as chemotherapy and hormonal therapies, are known to cause cerebral chemical changes, thus increasing the person’s risk of developing comorbid anxiety and depression [19]. Precise monitoring of all negative emotions, especially anxiety and depression, is an essential requirement in healthcare, because of their recognized interference and detrimental impact on the course and effectiveness of the overall treatment.

Schema Therapy proposes that some individuals may develop dysfunctional patterns of beliefs and unhelpful perceptions of the world and themselves [20, 21]. These early maladaptive cognitive patterns stand at the basis of psychological reactions that have developed over the course of our existence. Cognitive schemas are divided into distinct domains, such as abandonment/instability, mistrust/abuse, emotional inhibition, defectiveness/shame, social isolation/alienation, dependence/incompetence, vulnerability to harm or illness, enmeshment/undeveloped self, failure, entitlement/grandiosity, insufficient self-control/self-discipline, subjugation, self-sacrifice, approval-seeking/recognition-seeking, negativity/pessimism, unrelenting standards/hyper-criticalness, and punitiveness. In adults, these early maladaptive cognitive schemas are triggered by life-threatening events, such as the diagnosis of a neoplasm, and can lead to the development of emotions, such as fear, sadness, anger or shame.

The purpose of our study was to analyze the relationship between maladaptive cognitive schemas and the presence of anxiety and depression in women diagnosed with breast cancer. Also, we wanted to highlight the manner in which these schemas correlate with the decision to accept/refuse the breast reconstruction procedures offered, free of charge, by the Romanian National Health System for all patients that were subject to a mastectomy intervention.

Identifying maladaptive cognitive schemas, as well as symptoms of anxiety and depression, in women with breast cancer, helps tailor the psychotherapeutic interventions, while better supporting them in making an appropriate decision, but also in improving their overall quality of life.

## Material and methods

All patients included in this study (*N* = 64) were recruited from the „Pius Brînzeu” Timişoara County Emergency Clinical Hospital, „Casa Austria” Department of Plastic Surgery and Reconstructive Microsurgery, having been hospitalized throughout 2018 with a diagnosis of breast cancer. Due to the small number of subjects, the selection was based on inclusion/exclusion criteria, without the use of statistical methods.

*Inclusion criteria:*

1. A first admission in 2018 with a diagnosis of breast cancer, according to the World Health Organization’s International Statistical Classification of Diseases and Related Health Problems, 10th Revision (WHO ICD-10) criteria [22].
2. Women aged between 18 and 85 years.
3. Patients that accepted either a mastectomy-type of surgical intervention or a mastectomy, followed by breast reconstruction surgery.
4. All subjects have agreed to participate in the study.

*Exclusion criteria:*

1. Presence of intellectual disabilities.
2. Presence of a mental illness caused by psychoactive drugs or an organic disorder.
3. Patients hospitalized for a recurrence of cancer.

The entire sample (*N* = 64) was divided into two groups, depending on whether they accepted or refused to have subsequent breast reconstruction surgery. Group I consisted of 28 participants with breast cancer, who underwent mastectomy followed by breast reconstruction. Group II comprised 36 participants with breast cancer, who also underwent mastectomy, but refused to have subsequent breast reconstruction.

This study was approved by the Local Scientific Research Ethics Committee of the „Pius Brînzeu” Timişoara County Emergency Clinical Hospital.

### Assessments

After selecting the study group, both assessment scales were applied in the same session. The tools that we used were: a survey with general demographic questions, the Young Cognitive Schema Questionnaire - Short Form 3 (YSQ-S3) and the Romanian version of the 21 items Depression Anxiety Stress Scale, (DASS-21R). Both scales are free to use for academic purposes and have previously been validated for the Romanian population [23, 24].

We recruited all women with ages between 18 and 85 years, who were admitted for the purposes of surgical intervention due to a diagnosis of breast cancer, throughout the period between March–June 2018 (*N* = 64),

The general demographic questionnaire analyzed the following individual parameters: gender, age, home environment, educational level, and marital status. As per national guidelines, all other related medical and social history information regarding the menopausal status, body mass index, comorbidities, presence or absence of caregivers are recorded in the hardcopy file of each patient, stored in hospital archives. However, for the purposes of our study, this information has not been specifically quantified.

The YSQ - S3 contains 114 items representing various assertions and is marked on a 6-point Likert scale, where: 1 - completely untrue, 2 - most of the times untrue for me, 3 - in a certain measure rather true than untrue, 4 - moderately true for me, 5 - most of the times true for me, 6 - it describes me perfectly.

The DASS-21R scale includes 21 items, divided equally into three subscales (depression, anxiety, and stress), and requires the respondents to appreciate each item: 0 - did not apply to me at all; 1 - applied to me to some degree, or some of the time; 2 - applied to me to a considerable degree, or a good part of time; 3 - applied to me very much, or most of the time.

### Statistical analysis

The measured data were presented as mean and standard deviations for continuous variables with Gaussian distribution, median and interquartile range (IQR) for continuous variables without Gaussian distribution, or percentage (absolute frequency) for categorical variables. Continuous variable distributions were tested for normality by using the Shapiro–Wilk’s test, and for equality of variances by using Levene’s test.

To assess the significance of the differences between groups, the Student’s t-test (means, Gaussian populations), Mann–Whitney U test (medians, non-Gaussian populations), and Pearson chi-square or Fisher’s exact test (proportions) were used. The correlation between studied variables was evaluated using Spearman’s rank-sum correlation coefficient (non-Gaussian distributed variables), with its statistical significance having been assessed by using the t-distribution score test. The correlation coefficient always belongs to the interval [− 1, 1]. When its value is exactly − 1 or 1, it indicates a perfect correlation. A value of 0 indicates no correlation and the range of (0, 0.3) or (− 0.3, 0) indicates a weak correlation. A value around 0.5 (or − 0.5) indicates a moderate correlation, while a value greater than 0.7 (or lower than − 0.7) indicates a strong correlation.

Data were analyzed by using the Statistical Package for the Social Sciences v.17 software (SPSS Inc., Chicago, IL, USA). A *P*-value of 0.05 was considered as the threshold for statistical significance, and a confidence level of 0.95 was considered for estimating intervals.

## Results

The study sample included two distinct groups, namely, group I, consisting of 28 participants with breast cancer, who underwent a mastectomy followed by reconstructive breast surgery and group II, consisting of 36 participants with breast cancer, who also underwent a mastectomy, but refused to have subsequent reconstructive breast surgery (Table 1). Group I included participants aged between 29 and 76 years old (a median of 49 years old), while participants from group II had ages ranging between 40 and 84 years old (a median of 61.5 years, *p* < 0.001).

**Table 1:** Comparison of the socio-demographic data for the two groups of participants: Group I - with breast cancer, mastectomy, and breast reconstruction; Group II - with breast cancer, mastectomy, without reconstruction

	Group I	Group II	P
Number of participants	28	36	
Age [years]^a^	49.00 (43.50–56.00)	61.50 (55.50–67.00)	< 0.001^c^
Education			0.251^d^
High-school	14 (50%)	19 (50%)	
Post-high-school	2 (7.1%)	7 (19.4%)	
University Graduate/Master’s/Ph.D.	12 (42.9%)	10 (27.8%)	
Marital status			0.221^e^
Married	20 (71.4%)	25 (69.4%)	
Unmarried	4 (14.3%)	5 (13.9%)	
Divorced	4 (14.3%)	2 (5.6%)	
Widow	0 (0%)	4 (11.1%)	
Location [urban]^b^	22 (78.6%)	24 (66.7%)	0.294^d^

^a^ Continuous variables (without Gaussian distribution) are indicated by their median (inter-quartile range)

^b^ Categorical variables are presented by absolute frequency (percentage) in the sample

^c^ Independent samples Mann-Whitney U test (2-tailed)

^d^ Chi-square test (2-tailed)

^e^ Fisher’s exact test

The DASS-21 questionnaire, used to identify the level of depression, anxiety, and stress, showed significant differences between the two samples (Table 2). We were able to observe that participants from group I presented significantly lower levels of anxiety, as well as depression and stress. However, we noted that there were no significant differences between the proportion of participants displaying the three different intensities of depressive symptoms (normal, mild-moderate and severe-extremely severe) (Chi-square test X^2^ (2) = 1.679, *p* = 0.432). On the contrary, we observed that participants in group II presented significantly higher levels of anxiety than those who chose to benefit from breast reconstruction (Chi-square test, X^2^ (2) = 6.157, *p* = 0.046). In addition, we did not identify any significant differences between the proportion of participants, when considering their stress levels (Chi-square test, X^2^ (2) = 2.681, *p* = 0.261).

**Table 2:** Comparison between the two groups (Group I – patients who underwent mastectomy and breast reconstruction; Group II - mastectomy, without reconstruction) considering the depression, anxiety, and stress levels, as described by the DASS-21 test

DASS-21^a^	Group I	Group II	P^c^
*Depression*^*b*^			0.432
Normal	20 (71.40%)	29 (80.60%)	
Mild – Moderate	7 (25.00%)	7 (19.40%)	
Severe – Extremely severe	1 (1.60%)	0 (0%)	
*Anxiety*			**0.046**
Normal	20 (71.4%)	19 (52.80%)	
Mild – Moderate	4 (14.3%)	15 (41.70%)	
Severe – Extremely severe	4 (14.3%)	2 (5.60%)	
*Stress*			0.261
Normal	22 (78.6%)	31 (86.1%)	
Mild – Moderate	4 (14.3%)	5 (13.9%)	
Severe – Extremely severe	2 (7.1%)	0 (0%)	

^a^ DASS-21 contains three subscales, Depression, Anxiety, and Stress, each one including seven items; the score for each subscale ranges from 0 to 21

^b^ Data are presented as absolute frequency (percentage)

^c^ Chi-square test (2-tailed)

In terms of all of the cognitive schemas, scores in group I were lower than those in group II (Table 3). Moreover, we observed significant differences between the two groups, when considering the ‘enmeshment/undeveloped self’ (8.50 vs. 11.50, Mann-Whitney U test, p = 0.019) and ‘punitiveness’ (31.00 vs. 37.00, Mann-Whitney U test, *p* = 0.009) sub-scales.

**Table 3:** Comparison of the two groups considering the YSQ-S3 cognitive schemas

Cognitive schemas YSQ-S3^a^	Group I	Group II	P^b^
Emotional Deprivation^c^	8.00 (5.00–12.50)	10.00 (5.00–15.50)	0.675
Abandonment / Instability^c^	8.00 (6.00–11.00)	9.00 (5.50–13.50)	0.785
Mistrust / Abuse^c^	10.00 (7.50–13.00)	13.00 (9.00–15.50)	0.130
Social isolation / Alienation^c^	7.00 (5.00–10.50)	9.00 (5.50–12.50)	0.209
Defectiveness / Shame^c^	6.50 (5.00–9.00)	6.00 (5.00–9.00)	0.574
Failure^c^	8.00 (5.00–10.00)	10.00 (5.00–12.00)	0.254
Dependence / Incompetence^c^	9.00 (7.50–11.00)	9.50 (7.00–12.50)	0.908
Vulnerability to harm or illness^c^	7.00 (5.00–9.00)	7.00 (5.00–9.00)	0.983
Enmeshment / Undeveloped Self^c^	8.50 (5.50–11.00)	11.50 (9.00–14.50)	**0.019**
Subjugation^c^	11.00 (8.00–16.00)	12.00 (10.00–14.00)	0.887
Self-sacrifice^c^	19.00 (15.50–24.00)	21.50 (19.00–25.00)	0.081
Emotional inhibition^c^	10.00 (9.00–13.50)	11.00 (9.00–15.00)	0.458
Unrelenting Standards / Hypercriticalness^c^	16.00 (14.00–18.00)	16.50 (14.00–18.00)	0.827
Entitlement / Grandiosity^c^	11.00 (9.00–14.50)	13.00 (10.00–15.00)	0.154
Insufficient Self-Control / Self-Discipline^c^	9.50 (7.50–12.00)	10.50 (9.00–14.00)	0.279
Approval-Seeking / Recognition-Seeking^d^	35.00 (30.50–48.50)	39.00 (28.50–50.00)	0.882
Negativity / Pessimism^e^	19.50 (15.00–25.50)	21.00 (14.50–32.00)	0.630
Punitiveness^d^	31.00 (22.00–37.00)	37.00 (28.00–42.50)	**0.009**

^a^Data are presented by their median (inter-quartile range)

^b^Independent samples Mann-Whitney U test (2-tailed)

^c^Scale ranging from 0 to 30

^d^Scale ranging from 0 to 84

^e^Scale ranging from 0 to 66

The dominant schemas for the group of participants with breast reconstruction were: vulnerability to harm or illness, subjugation, self-sacrifice, and approval-seeking/recognition-seeking. In the case of the group of participants without breast reconstruction, the dominant schemas were: emotional deprivation, mistrust/abuse, failure, enmeshment/undeveloped self, self-sacrifice, unrelenting standards, negativity/pessimism, and punitiveness.

It should be noted that patients in both groups employed the self-sacrifice cognitive schema. We also noted that two cognitive schemas were not at all representative for the group of participants with breast reconstruction, namely, defectiveness/shame and enmeshment/undeveloped self.

We associated the sub-scale items of the YSQ-S3 test into five main cognitive schemas domains. For each domain, we studied its correlation with the levels of depression, anxiety, and stress for both groups. We observed that participants with breast reconstruction presented cognitive schemas, which did not result in depressive or anxiety symptoms. On the contrary, participants without breast reconstruction presented cognitive schemas that were significantly correlated with the presence of depressive or anxiety symptoms. For example, in the case of the cognitive schema domain of ‘disconnection and rejection’, its correlation with depressive symptoms was very weak and not significant for the group with breast reconstruction (Spearman’s *ρ* = 0.091, *p* = 0.644), while for the group without reconstruction, the correlation was significantly moderate-strong (Spearman’s *ρ* = 0.647, *p* < 0.01) (Table 4). In addition, in the case of the cognitive schema domain of ‘over vigilance and inhibition’, its correlation with depressive symptoms was very weak and not significant for the group with breast reconstruction (Spearman’s *ρ* = 0.098, *p* = 0.619), while for the group without reconstruction, the correlation was significantly moderate (Spearman’s *ρ* = 0.361, *p* = 0.030) (Table 4).

**Table 4:** The relation between cognitive schemas and depression (D), anxiety (A) and stress (S) for the two groups of participants: Group I - mastectomy, and breast reconstruction; Group II - mastectomy, without reconstruction

Cognitive schemas domains	Group I			Group II		
	D^a^	A^a^	S^a^	D^a^	A^a^	S^a^
Disconnection and rejection	0.091	0.307	0.328	0.647^c^	0.359^b^	0.614^c^
Impaired autonomy & performance	0.286	0.530^c^	0.628^c^	0.476^c^	0.329	0.284
Other-directedness	0.034	0.200	0.308	0.154	0.119	0.211
Over vigilance and inhibition	0.098	0.040	−0.095	0.361^b^	0.287	0.256
Impaired limits	0.198	−0.075	0.037	0.209	0.038	0.087
Approval-Seeking / Recognition-Seeking	0.081	0.194	0.196	0.205	0.064	0.039
Negativity / Pessimism	−0.018	0.156	0.385^b^	0.620^c^	0.481^c^	0.493^c^
Punitiveness	0.052	0.097	0.336	0.318	0.240	0.321

^a^Results are presented as Spearman’s correlation coefficient ρ

Notes: ^b^Correlation is significant at the 0.05 level (two tailed t-test); ^c^Correlation is significant at the 0.01 level (two tailed t-test).

Furthermore, we observed that, for the group of participants without breast reconstruction, the dominant cognitive schemas were significantly correlated with the presence of depression, anxiety, and stress. For example, emotional deprivation and mistrust/abuse, which are included in the cognitive domain of ‘disconnection and rejection’, were correlated with depression (Spearman’s *ρ* = 0.647, *p* < 0.01), anxiety (Spearman’s *ρ* = 0.359, *p* < 0.05) and stress (Spearman’s *ρ* = 0.614, *p* < 0.01). Also, the dominant cognitive schemas ‘unrelenting standards/hyper-criticalness’, included in the ‘over vigilance and inhibition’ domain, were significantly correlated with the presence of depression (Spearman’s *ρ* = 0.361, *p* < 0.05). In addition, the ‘negativity/pessimism’ schema was very significantly correlated with depression (Spearman’s *ρ* = 0.620, *p* < 0.01), anxiety (Spearman’s *ρ* = 0.481, *p* < 0.01) and stress (Spearman’s *ρ* = 0.493, *p* < 0.01).

**Fig. 1:**
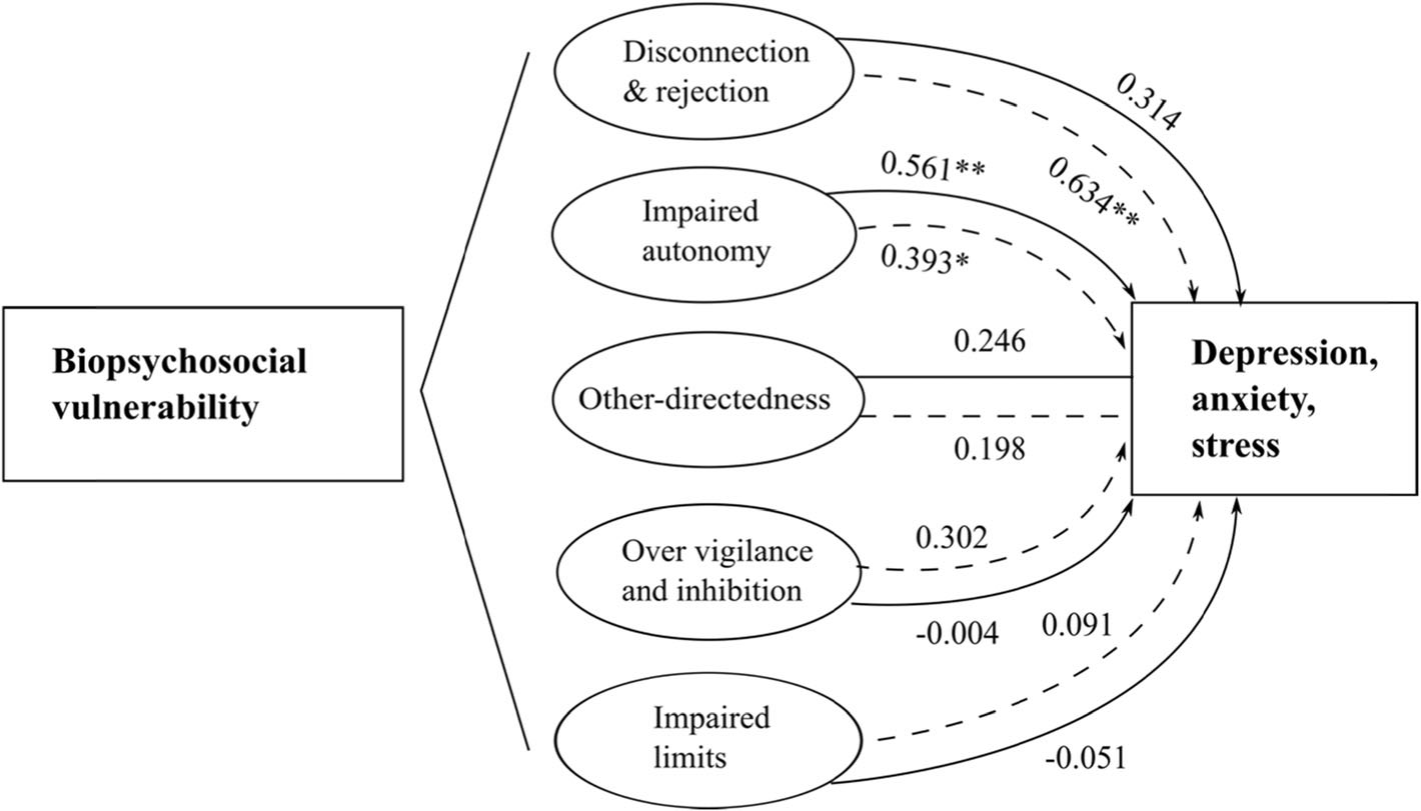
Representation of cognitive schemas’ influence on the mixed anxiety-depressive symptomatology. Note: Continuous line - participants with breast cancer, mastectomy, and breast reconstruction; Discontinuous line - participants with breast cancer, mastectomy, without reconstruction. *Notes:* *Correlation is significant at the 0.05 level (two-tailed t-test); **Correlation is significant at the 0.01 level (two-tailed t-test)

The influence of cognitive schemas on the mixed anxiety-depressive symptomatology in both groups of participants can be seen in Fig. 1. We highlighted that for participants in group I the cognitive schemas were less correlated with the presence of anxiety-depressive symptoms than for the group that refused breast reconstruction surgery. Moreover, in the latter case, the negative emotional schemas were significantly correlated with the presence of anxiety-depressive symptoms (Spearman’s *ρ* = 0.598, *p* < 0.001), while in the case of participants with breast reconstruction, the negative emotional schemas were not correlated with the presence of anxiety-depressive symptoms (Spearman’s *ρ* = 0.235, *p* = 0.229).

## Discussions

Currently, in Romania, there are up-to-date treatments that support people diagnosed with breast cancer to maintain an adequate quality of life and global functioning in accordance with their age. One of these treatments is represented by breast reconstruction following mastectomy, a procedure that is available for all Romanian women, via a national program that was started in 2014. However, the number of cases benefiting from this intervention in 2016 in Romania was of only 176 women [25]. Therefore, the following question arises: why is it that certain women diagnosed with breast cancer refuse to benefit from reconstructive procedures following a mastectomy? What lies behind this decision?

In this study, we have attempted to evaluate the relationship between cognitive schemas and the presence of anxiety and depression in women diagnosed with breast cancer, who have either accepted or refused to benefit from breast reconstruction surgery following a mastectomy. The correct identification of maladaptive cognitive schemas, as well as that of comorbid symptoms of anxiety and depression in this group of people, helps design specific psychotherapeutic interventions that aim to reduce mental health difficulties, by supporting patients when choosing the most appropriate individual treatment, as well as by helping to increase their overall quality of life [26, 27].

To date, the literature has focused on highlighting the clinical symptoms of depression and anxiety, and evaluating the role that these symptoms play in the evolution and prognosis of women with breast cancer. The maladaptive cognitive schemas proposed by Young have not been explored in women with breast cancer, although these are some of the key points from which the psychotherapeutic intervention is built.

The two patient groups that we evaluated in this study were reasonably homogenous, in terms of socio-demographic data; however, there were notable age differences between the two samples: the group with mastectomy and reconstruction surgery had an average age of 49 years compared to the other group of women, with an average age of 61.5 years. The other socio-demographic parameters were relatively similar, without significant differences.

The presence of clinical symptoms of anxiety, depression, and stress was identified by using the DASS-21 self-evaluation scale that performs an assessment based more on the dimensional, than on the categorical concept. The level of depression was noted to be similar in both groups, occurring in about one-quarter of all subjects and without any statistically significant differences. The same aspect was present when assessing levels of stress, even though some of these patients underwent reconstructive breast surgery. Differences only occurred in terms of anxiety levels, with higher scores found in group II, a fact that we correlated with the difficult decision process to not undergo reconstruction intervention. In general, decision-making is correlated with the frontal lobe and amygdala, from an anatomic viewpoint, but also with individual cognitive patterns [28–30].

The assessment of cognitive schemas was achieved by using the YSQ self-evaluation questionnaire - short form that showed that the group with mastectomy and reconstruction had lower values than the other group, and this while taking into account that we must have at least three responses with high scores (replies of 5 or 6) in order to conclude that there is a cognitive schema employed by the person. Even though this is a self-assessment questionnaire, it is essential that the results are discussed with the patients, which helps to practically connect their cognitive patterns to their current life problems. Results from this questionnaire showed significant differences between the two groups at the ‘enmeshment / undeveloped self’ (8.50 vs. 11.50, Mann-Whitney U test, *p* = 0.019) and ‘punitiveness’ (31.00 vs. 37.00, Mann-Whitney U test, *p* = 0.009) level, meaning that they were prominent in the group of patients who have not opted for reconstructive surgery. The ‘enmeshment / undeveloped self’ cognitive schema encompasses emotional involvement and excessive attachment to their significant others and is usually experienced as a sense of emptiness or a lack of direction, of existential meaninglessness. In a similar vein, the ‘punitiveness’ schema involves difficulties forgiving personal or others’ mistakes, as well as difficulties when faced with having to accept imperfections, and is experienced as a general sense of anger and intolerance. Both schemas can be correlated with the decision of mastectomy without reconstruction through a lack of existential purpose and the tendency to care for oneself in a harsh, punitive manner.

The dominant schemas for the group of participants with breast reconstruction were ‘vulnerability to harm or illness’, ‘subjugation’, ‘self-sacrifice’ and ‘approval-seeking’/‘recognition-seeking’.

The ‘vulnerability to harm or illness’ schema falls in the domain of ‘autonomy and poor performance’, while the other three types are in the field of ‘orientation towards others’. The domain of ‘autonomy and poor performance’ implies a lower ability to function independently, which translates into difficulties of leaving the family of origin and, also, of setting personal goals. The domain of ‘focusing on others’ involves maintaining emotional ties with other people and making efforts to avoid upsetting other people. Very often, these individuals will tend to focus more on social appearances than on their individual needs.

Another notable aspect from this study is that most of the schemas that we identified belong to the area of conditioned cognitive schemas that frequently develop in order to reduce the suffering caused by unconditioned schemas and to, somehow, help people cope with their life struggles [21].

The dominant schemas identified in the group of participants without breast reconstruction were: ‘emotional deprivation’, ‘mistrust/abuse’, ‘failure’, ‘enmeshment/undeveloped self’, ‘self-sacrifice’, ‘unrelenting standards’, ‘negativity/pessimism’ and ‘punitiveness’. The ‘unrelenting standards’, ‘negativity/pessimism’ and ‘punitiveness’ cognitive schemas belong to the category of ‘hypervigilance and inhibition’, while ‘emotional deprivation’, ‘mistrust/abuse’ and ‘enmeshment/undeveloped self’ are in the grouping of ‘separation and rejection’. The ‘failure’ schema is related to ‘autonomy and poor performance’. The existence of ‘hyper-vigilance’ schemas leads to the suppression of feelings, but also to a decrease in spontaneity and adherence to rigid internal rules. The domain of ‘separation and rejection’ includes cognitive schemas that cause the person to believe that fundamental needs, such as love, safety, and stability, will not be met.

It is also noteworthy that most of the schemas that we identified in this group of patients are unconditioned schemas, which, by definition, determine the person to believe that, no matter their choice, the end result will be the same.

Anyone who is faced with an adverse life event (e.g., receiving a diagnosis of breast cancer), which subsequently causes numerous unfavorable consequences, should contemplate all possible solutions and choose the one that they consider to be the best for their individual circumstances and needs. When making a decision, there are many factors involved, including the cognitive schemas described above. By analyzing the two samples of patients, we noticed some clear differences that could explain their opposite decisions regarding reconstructive surgery following a mastectomy. Cognitive schemas that focus on others are connected to the idea of maintaining an adequate emotional relationship with those around us and, as a result, the predominant idea is that everything should remain unchanged. For this category of patients, this goal was thought to be achievable by accepting to undergo reconstructive breast surgery.

Another aspect that we sought to analyze was the correlation of the five cognitive schema areas with clinical elements of depression, anxiety, and stress, and we attempted to achieve this by assessing all patients a few weeks after receiving the diagnosis, and also immediately after surgery. For the group of patients with mastectomy and no reconstructive surgery, there was a correlation between the early maladaptive schemas of ‘emotional deprivation’, ‘mistrust/abuse’ and ‘enmeshment/undeveloped self’ and anxiety and depressive symptoms (Spearman’s *ρ* = 0.598, *p* < 0.001). A potential explanation for this finding is that when someone experiences a significant or life-threatening illness and is faced with making critical decisions, the presence of these schemas, along with other factors, favors the occurrence of comorbid clinical depression and anxiety. However, in the group of patients who opted for breast reconstruction surgery, there were no correlations, and the clinical psychopathological symptoms were notably less prominent.

Schemas belonging to the area of ‘separation and rejection’ (‘defectiveness/shame’, ‘emotional deprivation’ and ‘social isolation’) that develop during childhood appear to be correlated with depressive elements in adult life. These models were found in the group of women who refused reconstructive surgery and were correlated with the presence of comorbid depressive and anxiety elements.

Cognitive schemas belonging to “impaired autonomy and performance” were correlated with anxiety and stress in the group of patients with mastectomy and reconstructive surgery, and with depression in the group of patients with mastectomy without reconstruction. Interestingly, we observed that patients with mastectomy and reconstructive surgery showed both anxiety and stress regarding vulnerability to harm or illness. An explanation is that the patients who opted for reconstruction are also exposed to human vulnerability. The patients with mastectomy and without reconstruction presented depressive elements regarding vulnerability to harm or illness, dependence/incompetence, enmeshment/undeveloped self and failure.

Another maladaptive schema that we identified was that of ‘subjugation’, by which a person conforms to the wishes of others, while repressing their own needs and desires. Surprisingly, this schema failed to correlate with any depressive elements, having only been identified and employed in the group of patients with mastectomy and reconstructive breast surgery. This result contradicts findings from other studies that describe an association of this schema with depression [31].

The ‘unrelenting standards/hyper-criticalness’ schema belongs to the ‘hypervigilance and inhibition’ area and consists of the belief that the person must meet high standards, in order to avoid criticism or punishment, thus being usually correlated with depressive clinical elements. This pattern was identified in the group of patients with mastectomy, without reconstructive breast surgery, and was significantly correlated with the presence of comorbid depression. Additionally, in the same sample of patients, the ‘negativity/pessimism’ schema was very significantly correlated with depression, anxiety, and stress; however, it is a well-known fact that this schema predisposes the person towards a constant focus on the negative aspects of life (death, negative things, unresolved problems, loss) and it is only natural to expect that a critical illness will re-activate it.

The anxious-depressive symptomatology correlates with certain cognitive schemas in the group of patients that refused reconstructive breast surgery; however, the issue that arises is related to the role they play in the onset of this symptomatology, but also to the role in the decisional process, namely whether or not to accept the second surgical intervention. Therefore, we suggest that it is essential to offer a psychiatric evaluation to all breast cancer patients, in order to exclude depressive and anxiety symptoms that may negatively influence the individual treatment choice, but also to identify early maladaptive cognitive schemas, so that a more adequate psychotherapeutic approach may be developed. We should also mention that, after receiving a diagnosis of breast cancer, patients are informed of their therapeutic options, thus having the possibility of individual choice. There were several limitations to this study, including the small sample size and the fact that we were only able to use self-assessment scales, which meant that these could have easily been influenced by the patients’ personality traits, their educational level or their family situation.

## Conclusions

The study revealed that there are significant correlations between certain early maladaptive schemas, such as ‘early emotional deprivation’, ‘mistrust/abuse’ and ‘enmeshment/undeveloped self’ and depressive and anxiety features in the category of patients with mastectomy, who refused to also undergo subsequent reconstructive breast surgery. These schemas belong to the field of ‘separation and rejection’ that correlate with a depressive pathology. The presence of these schemas, associated with anxiety and depressive elements, can influence the decisional process of these women, translating into a refusal of subsequent plastic surgery interventions, thus partly explaining the extremely low percentage of patients offered this auxiliary treatment option, despite its wide availability via a free-of-charge national program.

Future studies should be focusing on understanding the motivation that lies behind these decisions, so that local and national psychotherapeutic and psychoeducational programs can be developed, in order to further help this patient group. The primary purpose of these programs should be decreasing anxious and depressive symptoms and increasing the number of women who choose to benefit from breast reconstruction surgery after a mastectomy.

Furthermore, we suggest that regular clinical programs that seek to identify early maladaptive schemas can help tailor psychotherapeutic interventions that ultimately aim to maintain the premorbid quality of life of more patients diagnosed with breast neoplasms.

## Data Availability

The datasets used and/or analyzed during the current study are available from the corresponding author on reasonable request.

## Abbreviations

DASS-21R: Depression Anxiety Stress Scale, 21 items, Romanian version
IQR: Interquartile Range
SPSS: Statistical Package for the Social Sciences software
WHO ICD-10: World Health Organization 10th revision of the International Statistical Classification of Diseases and Related Health Problems
YSQ-S3: Young Schema Questionnaire 3 Short Form
